# Arsenic Internally and Subcutaneously in the Treatment of Lymphoma

**Published:** 1882-11-20

**Authors:** 


### ARSENIC INTERNALLY AND SUBCUTANEOUSLY IN THE TREATMENT OF LYMPHOMA.

A woman of sixty-five had difficulty in swallowing and breathing, and suffered from general feebleness, deafness, etc. Her condition was cachectic. Examination revealed a tumor in the posterior pharynx, filling up the nasal and pharyngeal cavities. The submaxillary and axillary glands were also swollen and hard. These growths were made to disappear, and the woman was regarded as cured in five months. This remarkable result was accomplished by the combined internal and parenchymatous administration of Fowler's solution. The arsenic was given in large doses, mixed with acetated tincture of iron, from eight to twenty-five drops three times a day. In this way twenty-eight grams were consumed in the course of the treatment. The injections consisted of equal parts of Fowler's solution and distilled water, of which there was injected from one to three tenths of the capacity of a Pravaz syringe (about three to nine minims.) There was but little reaction of the general organism, but a marked acceleration of the pulse. Locally, the tumors increased considerably in size with the first injections, but after the second week rapidly declined.—*Berl. Klin. Woch.*

[Czerny has employed the method of Billroth described above in the cure of a glandular lymphomata. In six months he obtained a complete cure of a case in which the patient had taken seven hundred and forty-six drops and had received seventy-six injections of ten drops each.—*Wien. Med. Wochen.; Michigan Medical News.*]

